# Supplementary material for: Local structural preferences in shaping tau amyloid polymorphism
Source: Nat Commun. 2024 Feb 3;15:1028. doi: 10.1038/s41467-024-45429-2 (PMC10838331; doi:10.1038/s41467-024-45429-2)
Supplement: Supplementary file 1 — Supplementary Information [file 41467_2024_45429_MOESM1_ESM.pdf]

Supplementary Information

Local Structural Preferences in Shaping Tau Amyloid Polymorphism

Nikolaos Louros[1,2], Martin Wilkinson[3], Grigoria Tsaka[1,2], Meine Ramakers[1,2], Chiara Morelli[1,2], Teresa Garcia[1,2], Rodrigo U. Gallardo[3], Sam D'Haeyer[4,5], Vera Goossens[4,5], Dominique Audenaert[4,5], Dietmar Rudolf Thal[6,7], Ian R. Mackenzie[8], Rosa Rademakers[9,10], Neil A. Ranson[3], Sheena E. Radford[3], Frederic Rousseau[1,2]*, Joost Schymkowitz[1,2]*

[1]Switch Laboratory, VIB Center for Brain and Disease Research, Herestraat 49, 3000 Leuven, Belgium

[2]Switch Laboratory, Department of Cellular and Molecular Medicine, KU Leuven, Herestraat 49, 3000 Leuven, Belgium

[3]Astbury Centre for Structural Molecular Biology, School of Molecular and Cellular Biology, University of Leeds, Leeds, LS2 9JT, UK

[4]VIB Screening Core, Ghent, Belgium

[5]Centre for Bioassay Development and Screening (C-BIOS), Ghent University, Ghent, Belgium

[6]KU Leuven, Leuven Brain Institute, 3000 Leuven, Belgium

[7]Laboratory for Neuropathology, KU Leuven, and Department of Pathology, UZ Leuven, 3000 Leuven, Belgium

[8]Department of Pathology and Laboratory Medicine, University of British Columbia, Vancouver, Canada.

[9]Applied and Translational Neurogenomics, VIB Center for Molecular Neurology, VIB, Antwerp, Belgium

[10]Department of Biomedical Sciences, University of Antwerp, Antwerp, Belgium

This PDF file includes:
Supplementary Figures 1-18
Supplementary Tables 1-2

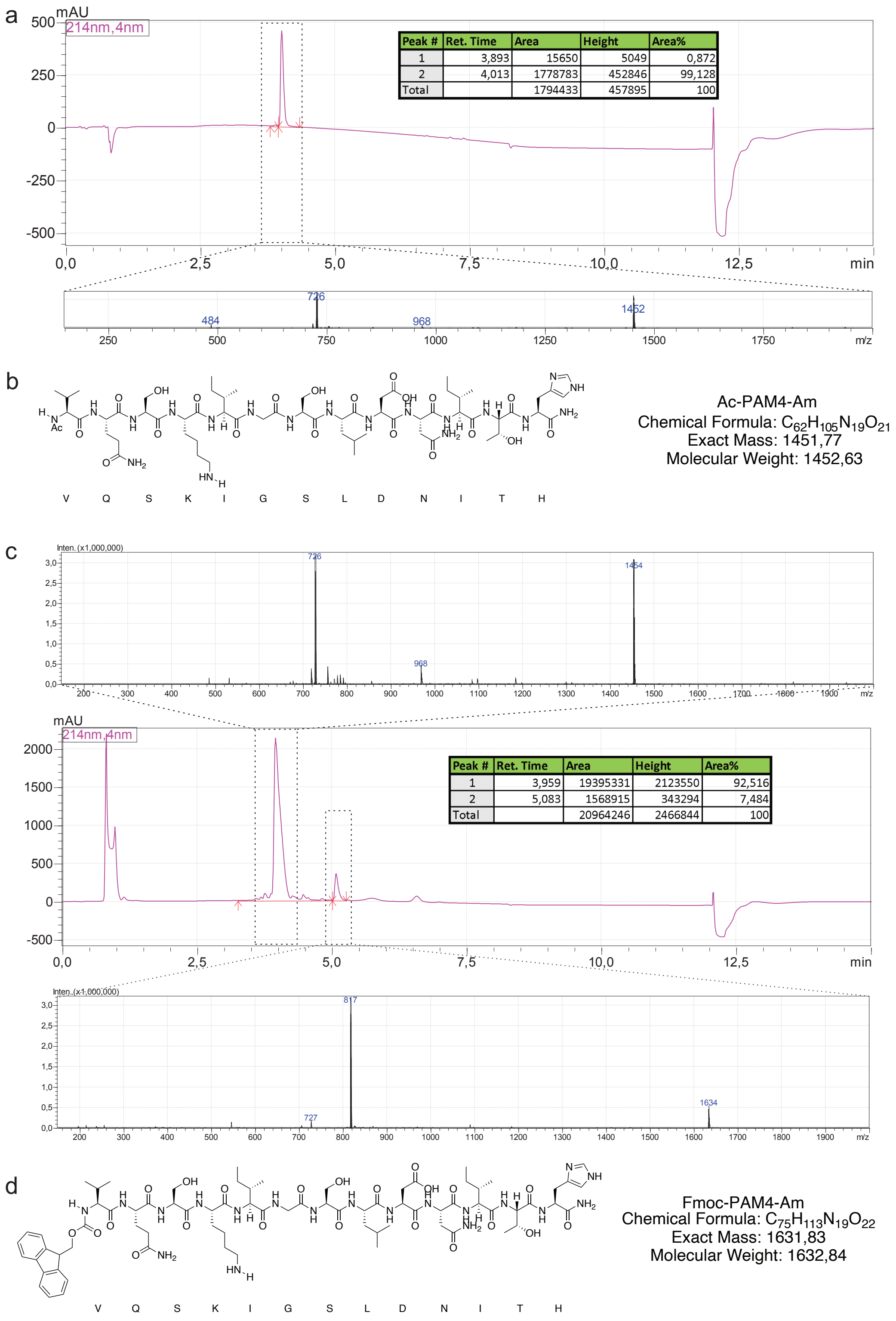

Supplementary Figure 1. PAM4 peptide quality control using HPLC and LCMS. (a) Chromatogram of a high purity N-terminally acetylated and C-terminally amidated PAM4 peptide batch (Ac-PAM4-Am). A single peak is identified (>99%), as shown in the inlet table, and mass spectrometry analysis matches the expected peptide molecular weight (b). (c) Chromatogram of a lower purity PAM4 peptide batch. A primary peak is identified (92.5%), matching the expected molecular weight of Ac-PAM4-Am, in addition to a second peak (7.4%) matching the mass of the protected peptide with an Fmoc adduct (Fmoc-PAM4-Am) (d).

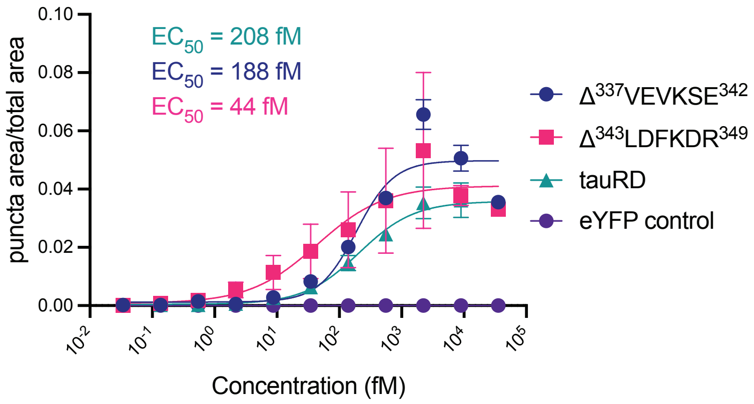

Supplementary Figure 2. Cellular screening assay of control constructs. Dose-response curves after the treatment of cells expressing tauRD (WT), Δ^{337}VEVKSE^{342}, Δ^{343}LDFKDR^{349,} or only the eYFP tag with various concentrations of extracts isolated from an AD patient (AD4 sample, see Table S1). Individual points represent mean values ± SEM (n=3 independent experiments). Curve fitting revealed minimal differences when comparing cells expressing the intact tauRD to those containing the Δ^{337}VEVKSE^{342} construct, and a slight increase in seeding efficiency in the Δ^{343}LDFKDR^{349} cells. As expected, the eYFP expression construct does not produce inclusions, validating the specificity of the seeding assay.

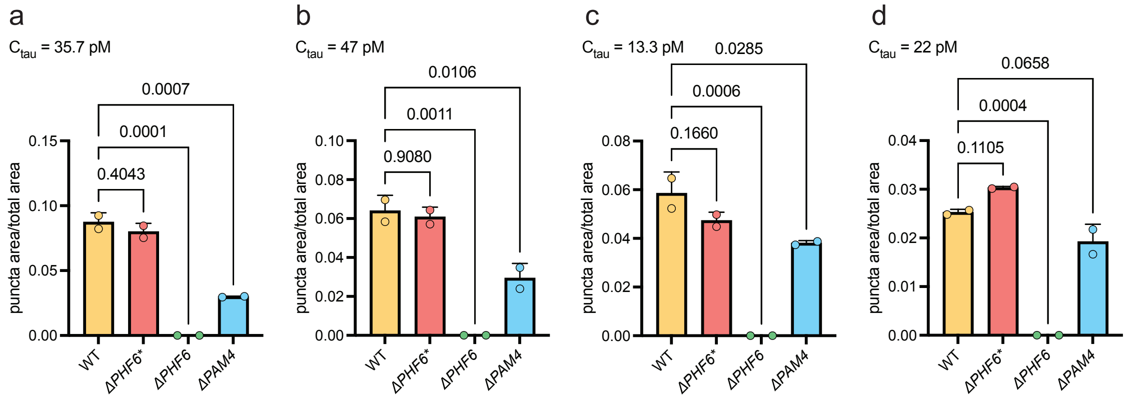

Supplementary Figure 3. Screening tau aggregates derived from various tauopathies with tauRD deletion constructs. Quantification of cellular seeding after the treatment of cells expressing tauRD (WT), ΔPHF6*, ΔPHF6, or ΔPAM4 with a single concentration (shown at the top of each graph) of tau seeds isolated from the brains of a patient diagnosed with (a) AD (AD4, see Table S2), (b) PSP, (c) CBD and (d) PiD. Bar plots represent mean values ± SD (n=2 independent experiments). Statistical significance was determined using one-way ANOVA with Dunnett's correction for multiple comparisons.

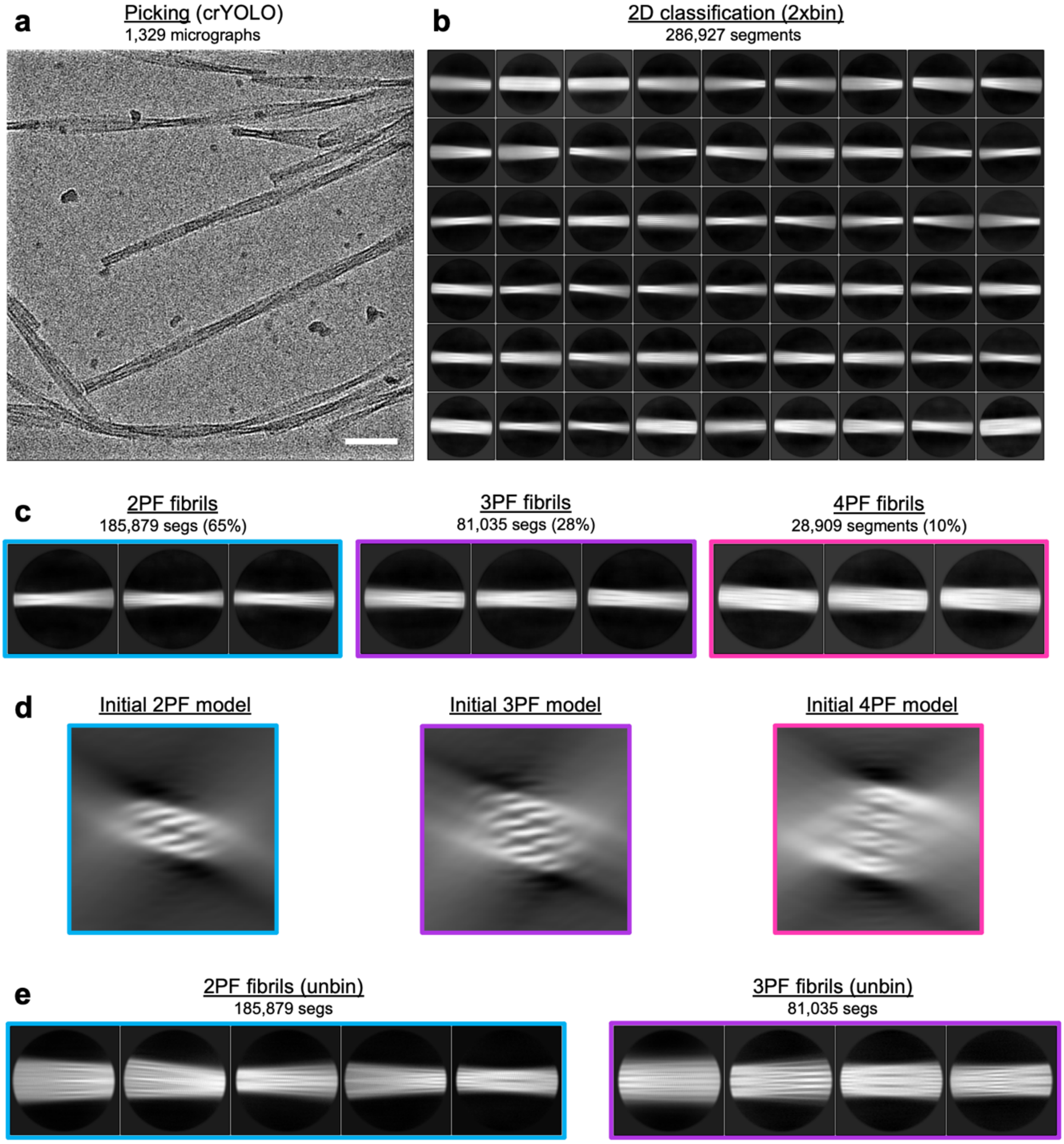

Supplementary Figure 4. Initial processing results from the high purity (>99%) PAM4 peptide cryoEM dataset. (a) Representative micrograph imaged with a defocus of 2.5 µm. The scale bar corresponds to 50 nm. (b) 2D class averages generated from the 2xbinned segment dataset after removing non-fibrillar picking artefacts, sorted row-by-row by population distribution with the most populated classes shown in the top left. The box dimensions are 57 nm for each class. (c) Selected, representative 2xbinned 2D class averages after splitting the data into three subsets based on fibril morphology, 2-protofilament (2PF), three-protofilament (3PF), and four-protofilament (4PF). (d) Slices of initial models for each morphology, generated from single 2D class averages with approximations of each crossover measured from the micrographs. These initial models were subsequently used as starting templates for 3D classification (see Supplementary Fig. 2). (e) Selected, representative 2D class averages from unbinned segments of the 2PF and 3PF subsets respectively.

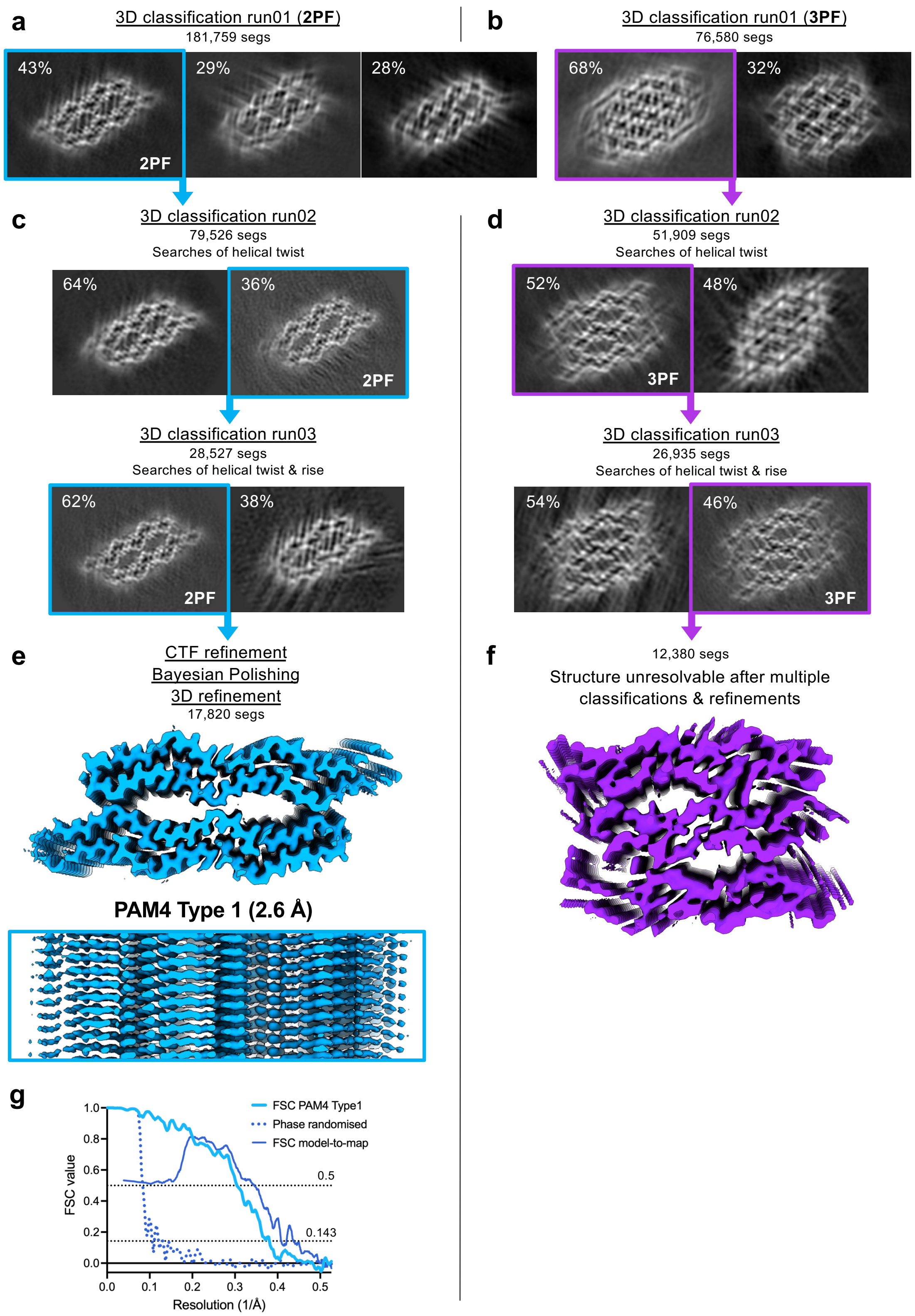

Supplementary Figure 5. Structure determination steps the high purity (>99%) PAM4 peptide cryoEM dataset. (a) Results of the first round of 3D classification with the 2PF segment subset. For each and subsequent 3D class averages, the averaged central 6x slices (representing a single helical layer) of each output map is displayed. (b) Results of the first round of 3D classification with the 3PF segment subset. (c) Results of subsequent rounds of

3D classification with the 2PF segment subset, selected classes proceeding to the next stage of processing are boxed in blue. (d) Results of subsequent rounds of 3D classification with the 3PF segment subset, selected classes proceeding to the next stage of processing are boxed in purple. (e) The centre of the final postprocessed, sharpened map of the 2PF fibril form determined at 2.6 Å resolution (0.143 FSC) is displayed with a boxed perpendicular view showing clear separation of β-strands. (f) The 3PF structure could not be resolved after multiple classifications and searches of helical twist and symmetry. The best-refined map is shown with incomplete backbone density. (g) FSC curve (for the gold-standard refined halfmaps) for the determined high purity PAM4 2PF fibril structure (PAM4 Type 1), with phase randomised FSC curve shown and the 0.143 FSC threshold shown as dotted lines.

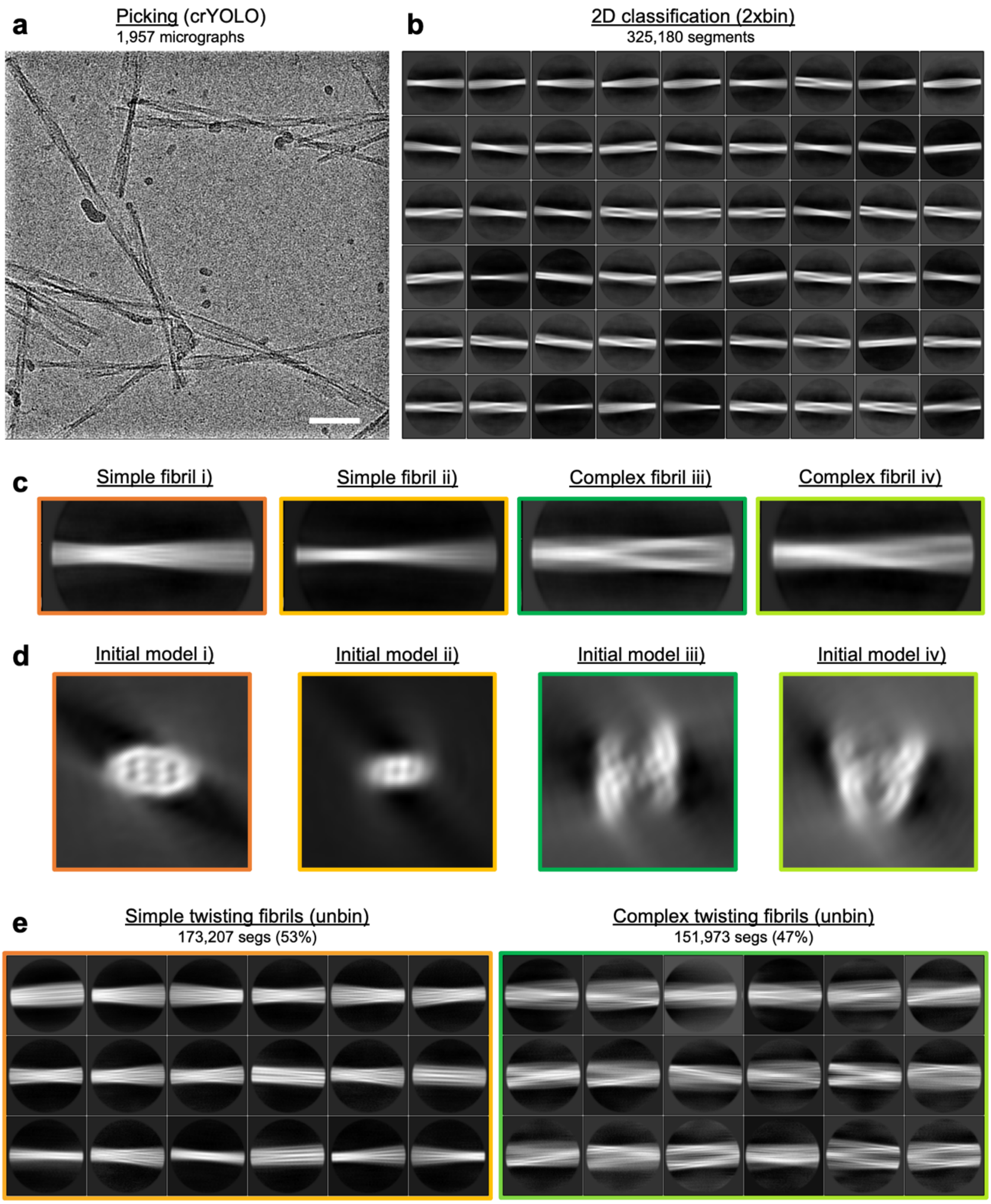

Supplementary Figure 6. Initial processing results from the lower purity (>92%) PAM4 peptide cryoEM dataset. (a) Representative micrograph, imaged with a defocus of 2.6 µm. The scale bar corresponds to 50 nm. (b) 2D class averages generated from the 2xbinned segment dataset after removing non-fibrillar picking artefacts, sorted row-by-row by population distribution with the most populated classes shown in the top left. The box dimensions are 56 nm for each class. (c) Selected, representative 2xbinned 2D class averages for each of the four potential fibril morphologies seen in the classification. (d) Slices of initial models for each potential morphology, generated from single 2D class averages with approximations of each crossover measured from the 2D class averages. The initial models i)

and iii) were subsequently used as starting templates for 3D classification (see Supplementary Fig. 4). (e) Selected, representative 2D class averages from unbinned segments after splitting the data into two subsets based on fibril morphology.

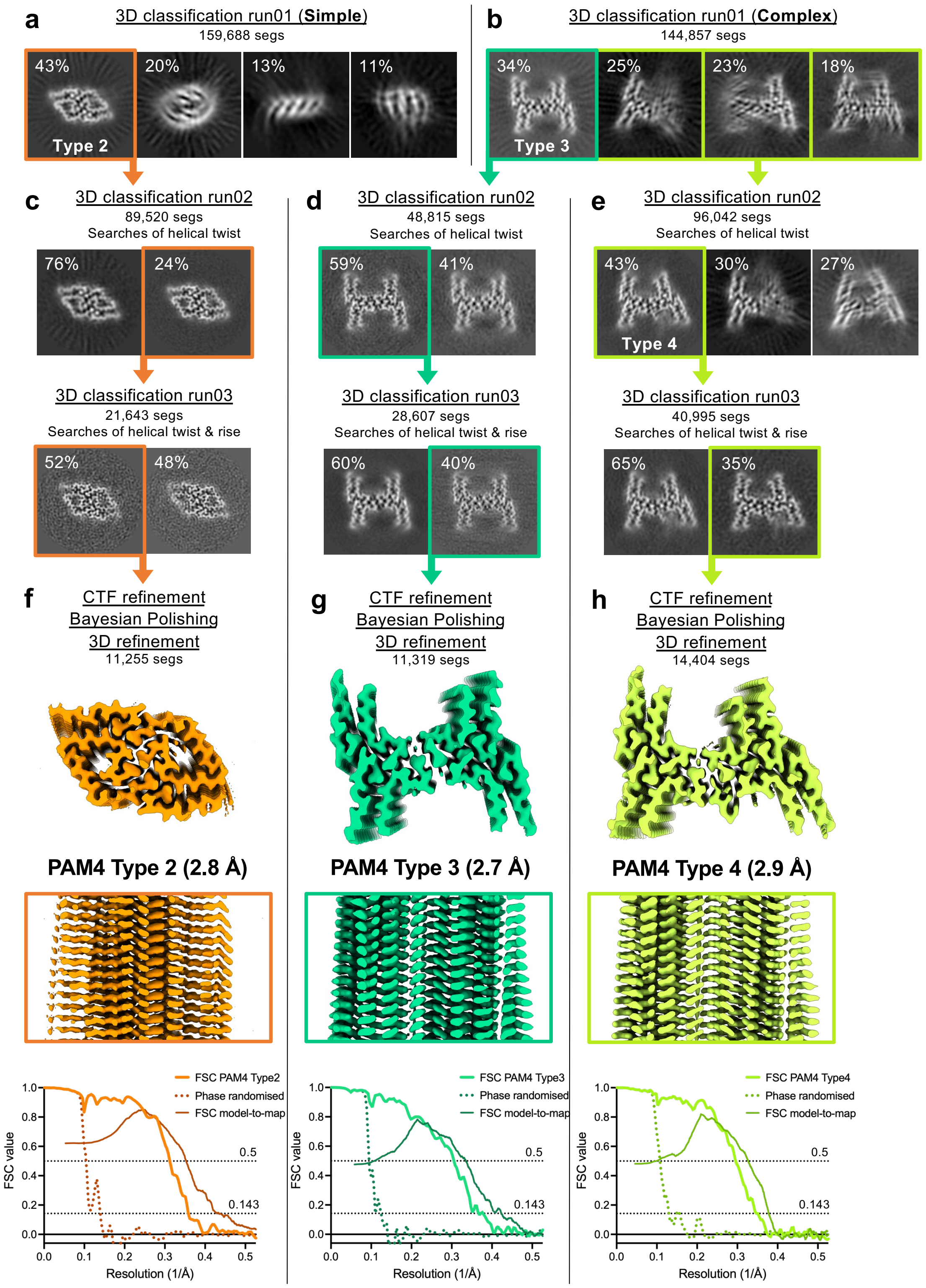

Supplementary Figure 7. Structure determination steps the lower purity (>92%) PAM4 peptide cryoEM dataset. (a) Results of the first round of 3D classification with the simple segment subset. For each and subsequent 3D class averages, the averaged central 6x slices (representing a single helical layer) of each output map is displayed. (b) Results of the first round of 3D classification with the complex segment subset. (c-e) Results of subsequent rounds of 3D classification with the Type 2, Type 3, and Type 4 fibril segments. Selected classes proceeding to the next stage of processing are boxed in orange, green and lime green

for Type 2, 3 and 4 fibril segments, respectively. (f) The centre of the final postprocessed, sharpened map of the Type 2 fibril determined at 2.8 Å resolution (0.143 FSC) is displayed with a boxed perpendicular view showing clear separation of β-strands. Below is the FSC curve (for the gold-standard, refined halfmaps) and phase randomised FSC curve for the same structure with the 0.143 FSC threshold shown as dotted lines. (g) The centre of the final postprocessed, sharpened map of the Type 3 fibril determined at 2.7 Å resolution (0.143 FSC) is displayed with a boxed perpendicular view showing clear separation of β-strands and the corresponding FSC curves as described previously. (h) The centre of the final postprocessed, sharpened map of the Type 4 fibril determined at 2.9 Å resolution (0.143 FSC) is displayed with a boxed perpendicular view showing clear separation of β-strands and the corresponding FSC curves as described previously.

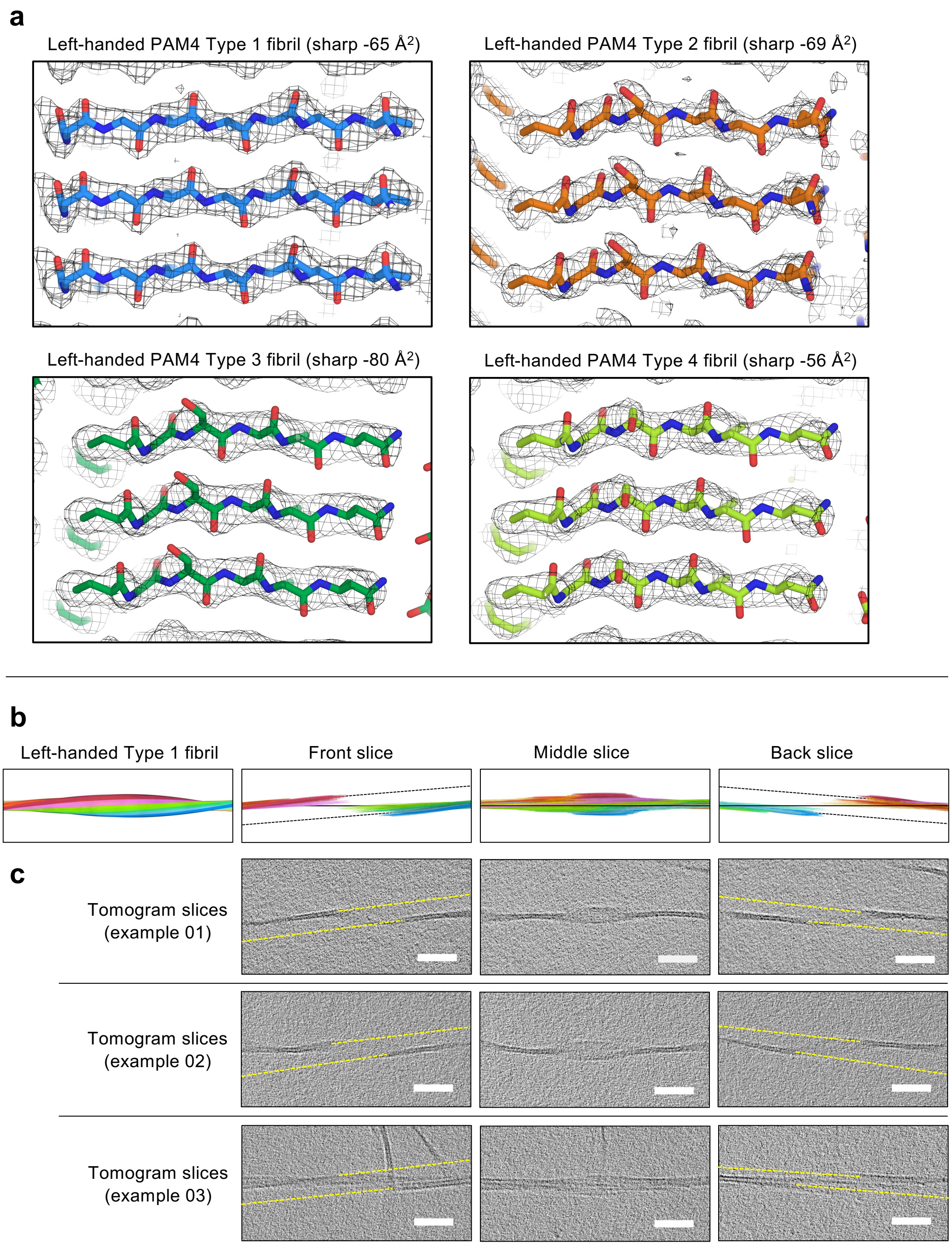

Supplementary Figure 8. Fibril hand determination using the resolution of the cryoEM maps and cryoET reconstruction supports left-handed PAM4 fibrils. (a) Representative sections of the cryoEM maps (grey mesh) with fitted, left-handed peptide models shown as sticks for each of the solved PAM4 fibril structures. The maps support left-handed models based on the orientation and fit of the peptide backbone. (b) Surface view of the PAM4 Type 1 fibril structure expanded along the length of one helical crossover, modelled as left-handed. (c) Slices of the front/top, middle, and back/bottom of each fibril structure are displayed for identification of twist direction in the corresponding tomograms. Slices through the front/top (late z-slice), middle (middle z-slice), and back/bottom (early z-slice) of three different, single

fibrils in cryoET tomograms collected on the same PAM4 cryoEM grid as used for structure determination of fibril Type 1. The scale bar represents a distance of 50 nm and dotted yellow track lines are drawn for reference to show the direction of the twisting protofilaments. Each image corresponds to single z-slices with thicknesses of 5.8 Å. CryoET processing of a control fibril sample of known hand (corroborated by AFM imaging)[1] was used to establish a processing pipeline that generates tomograms with the correct hand using the same microscope and collection strategy deployed for these tomograms of PAM4 fibrils (as described in the methods).

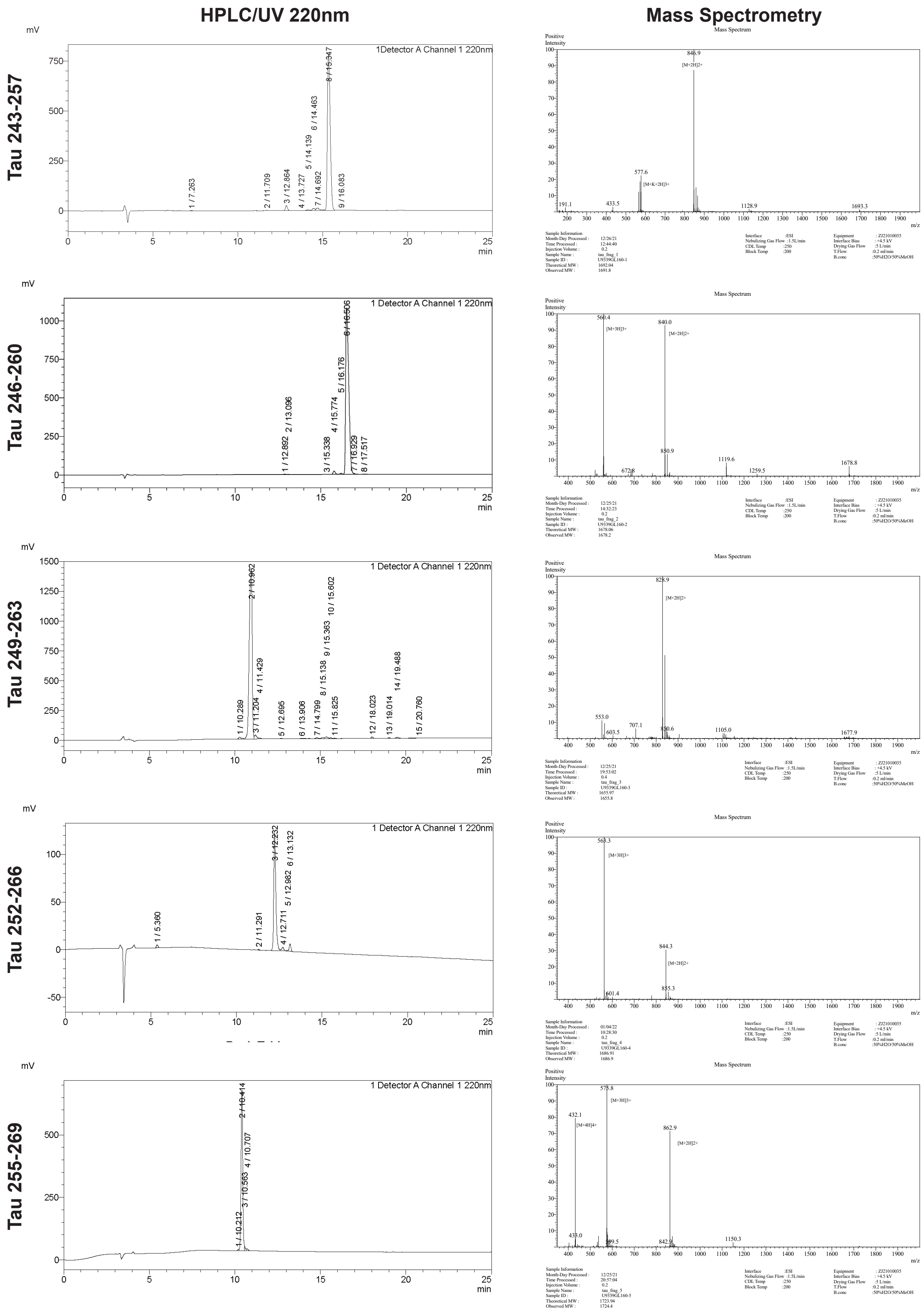

Supplementary Figure 9. Peptide quality control. HPLC chromatograms (left) and mass spectrometry (right) for peptides spanning tauRD residues 244-269.

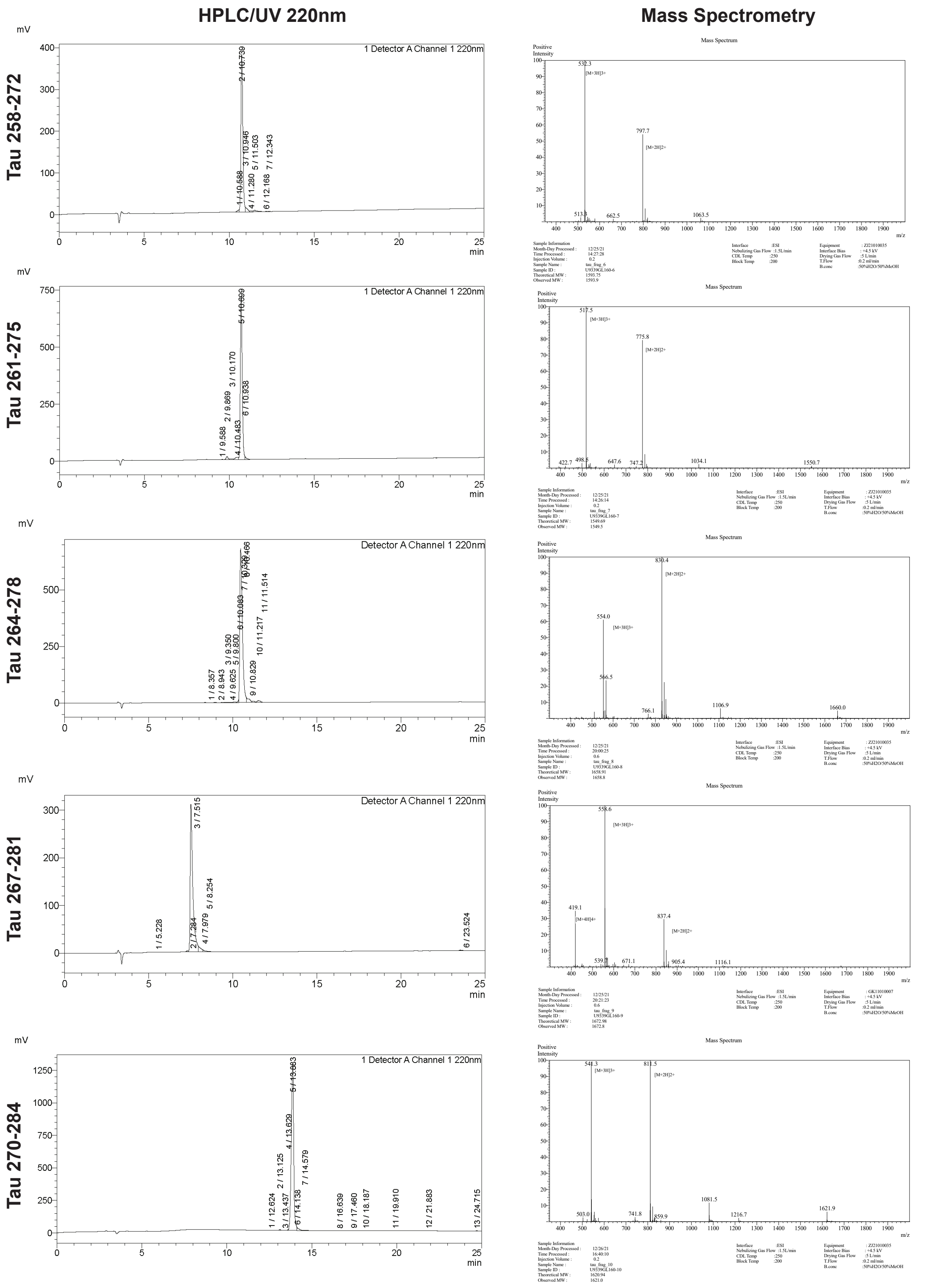

Supplementary Figure 10. Peptide quality control. HPLC chromatograms (left) and mass spectrometry (right) for peptides spanning tauRD residues 258-284.

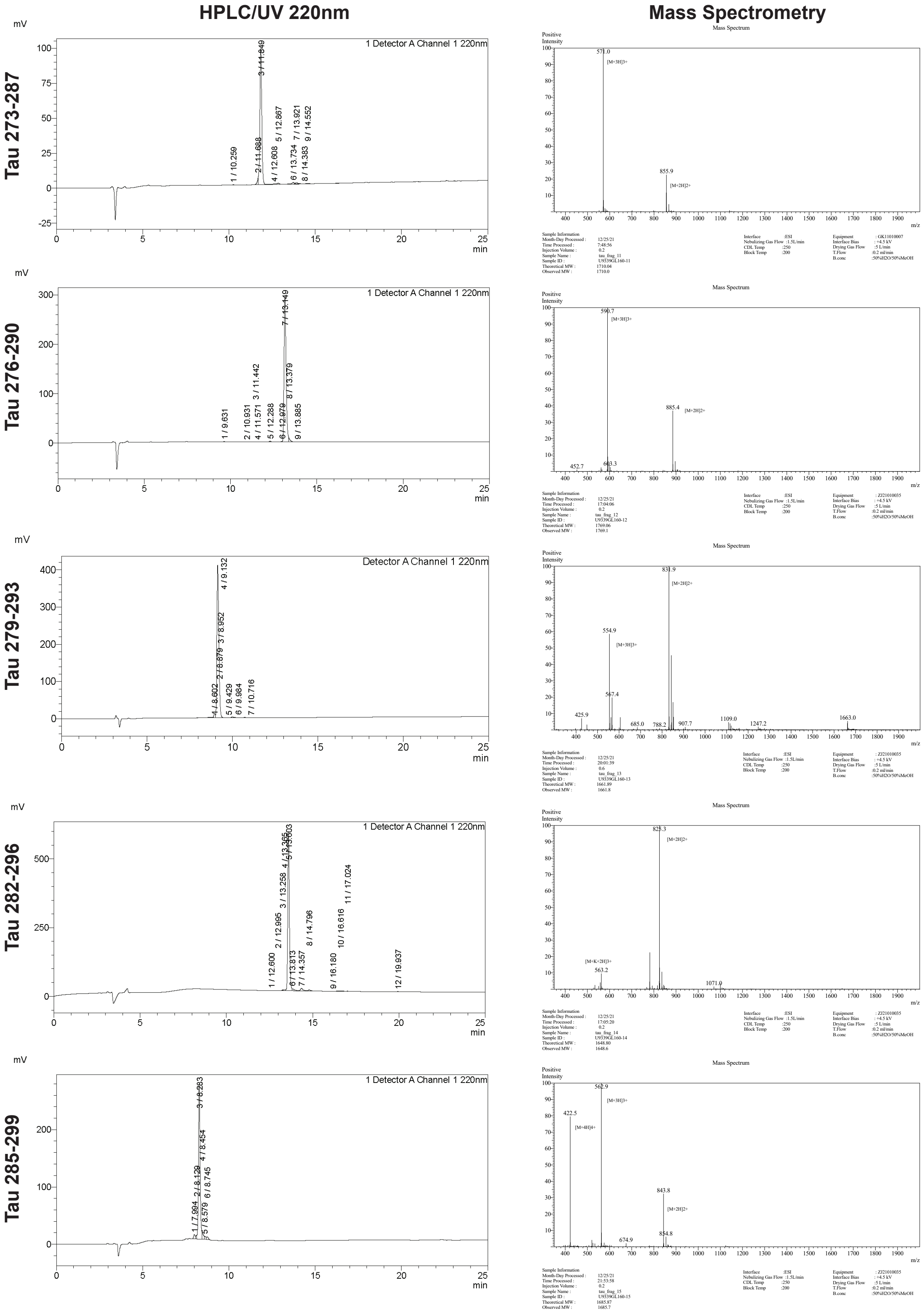

Supplementary Figure 11. Peptide quality control. HPLC chromatograms (left) and mass spectrometry (right) for peptides spanning tauRD residues 273-299.

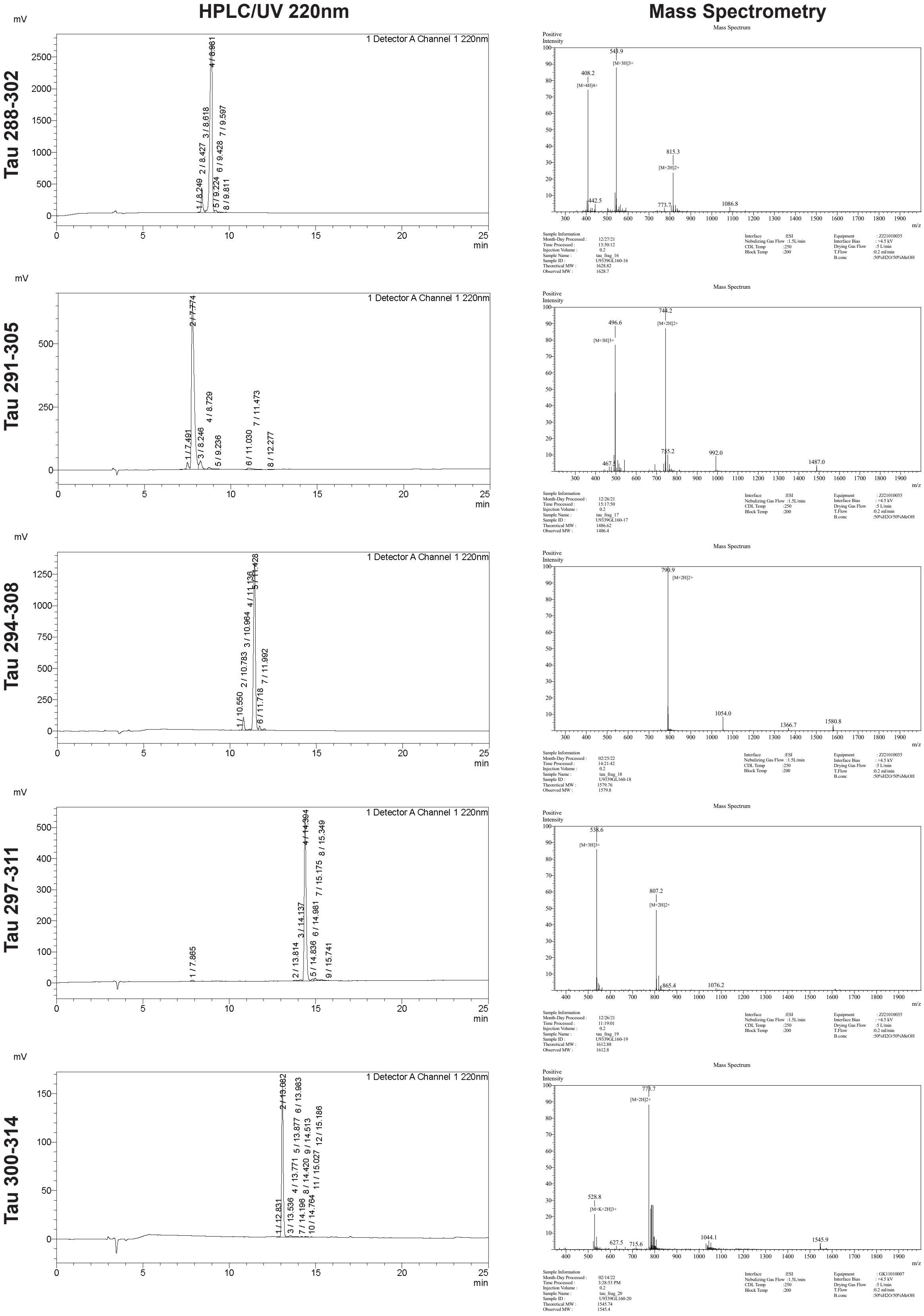

Supplementary Figure 12. Peptide quality control. HPLC chromatograms (left) and mass spectrometry (right) for peptides spanning tauRD residues 288-314.

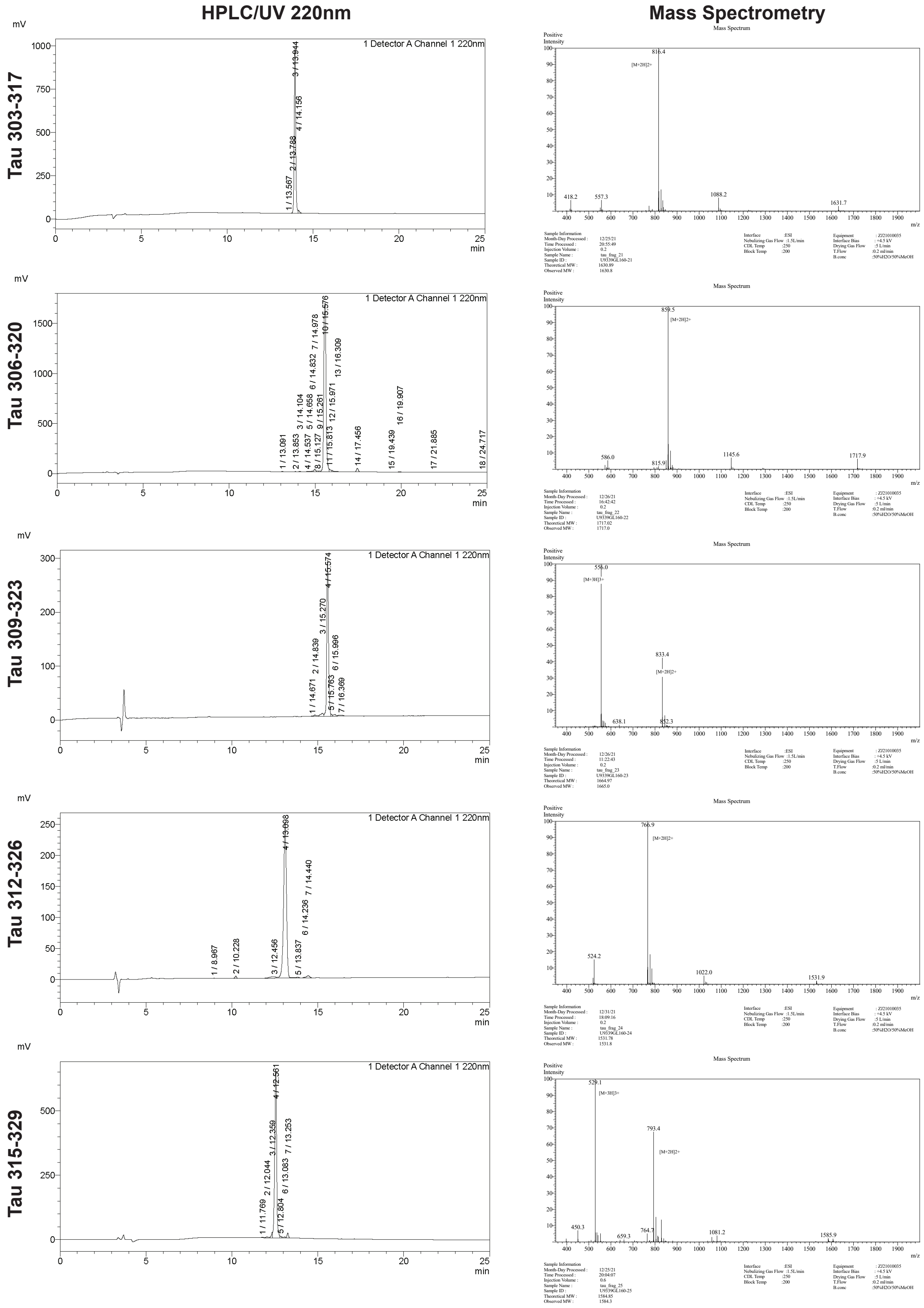

Supplementary Figure 13. Peptide quality control. HPLC chromatograms (left) and mass spectrometry (right) for peptides spanning tauRD residues 303-329.

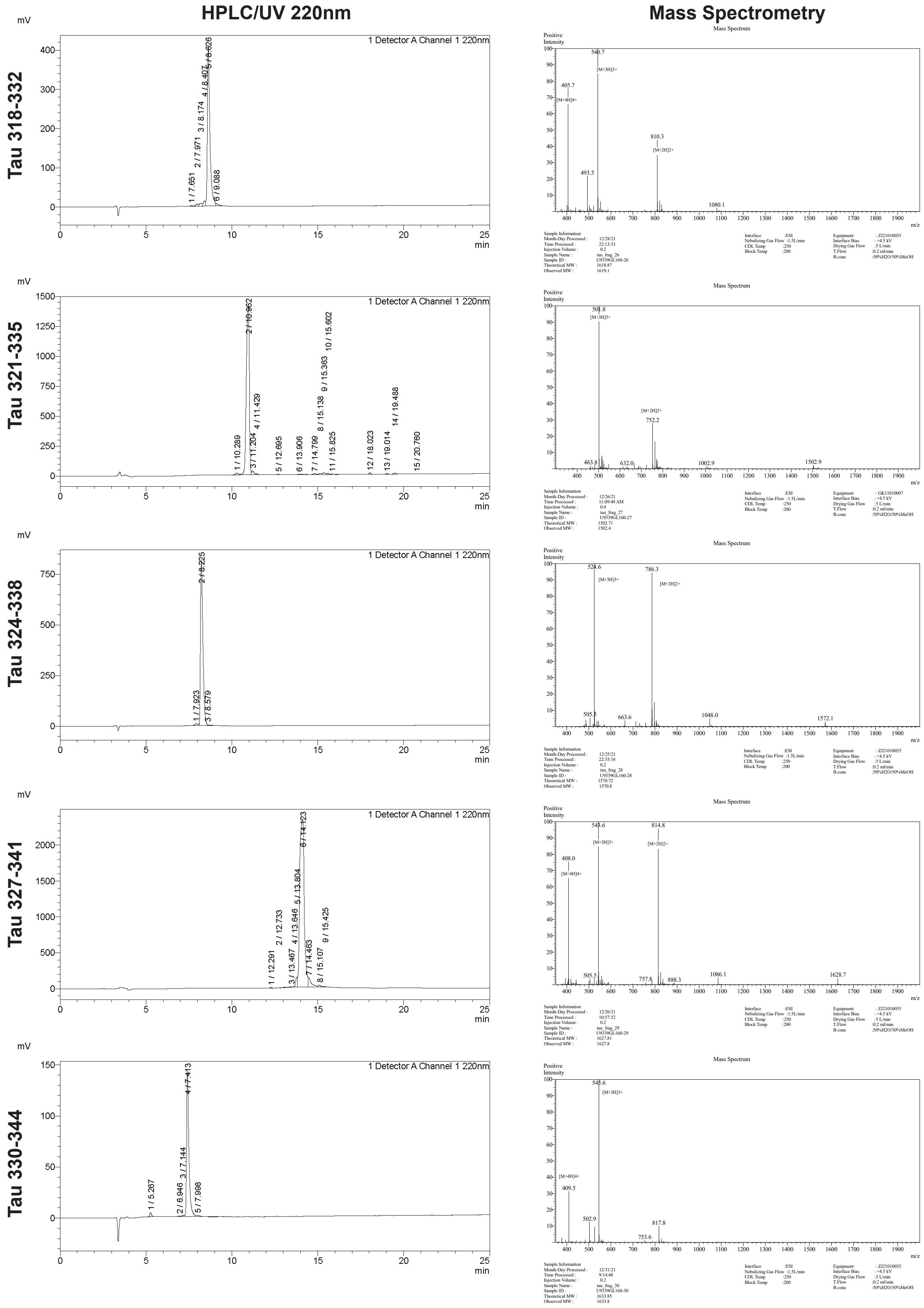

Supplementary Figure 14. Peptide quality control. HPLC chromatograms (left) and mass spectrometry (right) for peptides spanning tauRD residues 318-344.

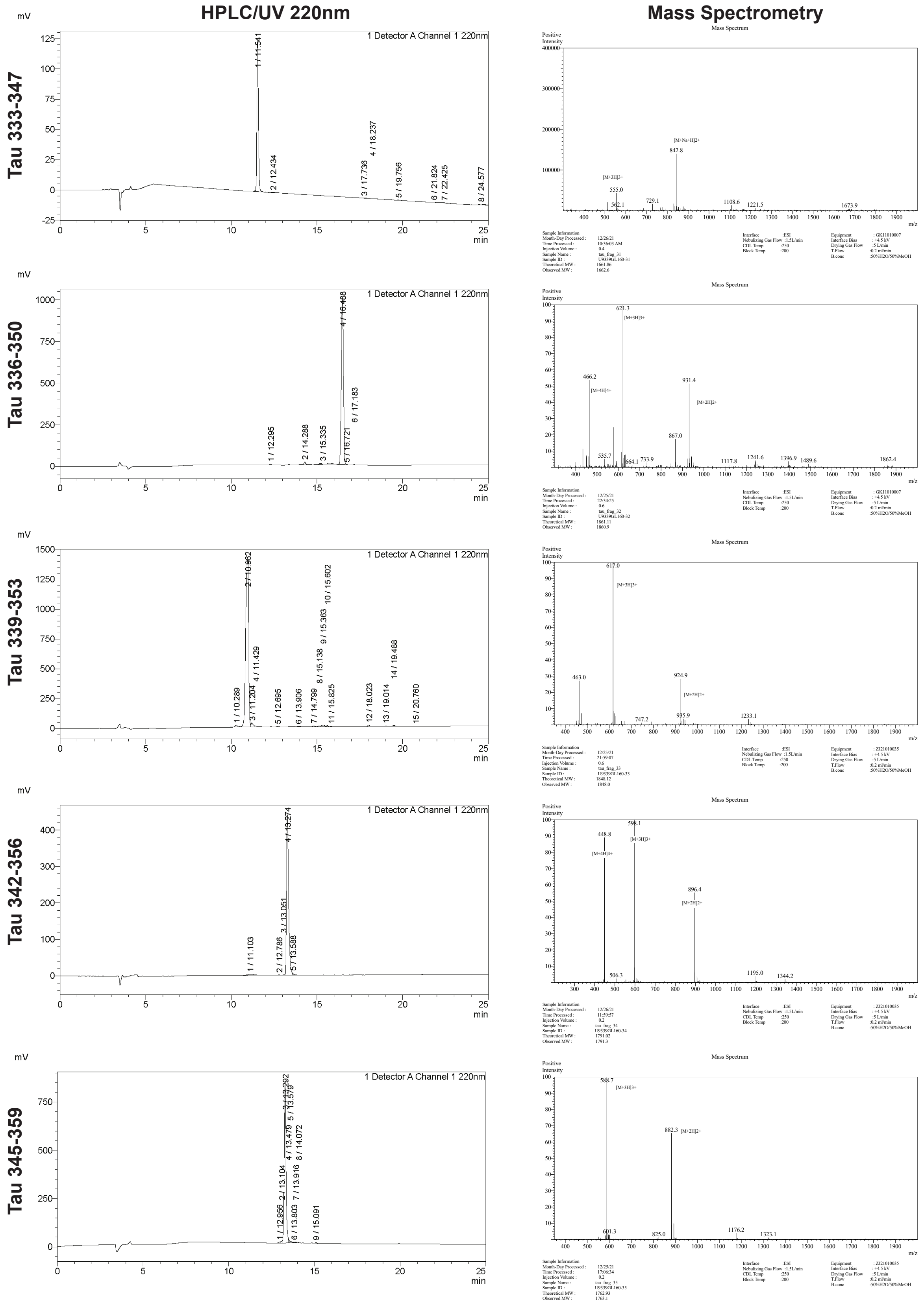

Supplementary Figure 15. Peptide quality control. HPLC chromatograms (left) and mass spectrometry (right) for peptides spanning tauRD residues 333-359.

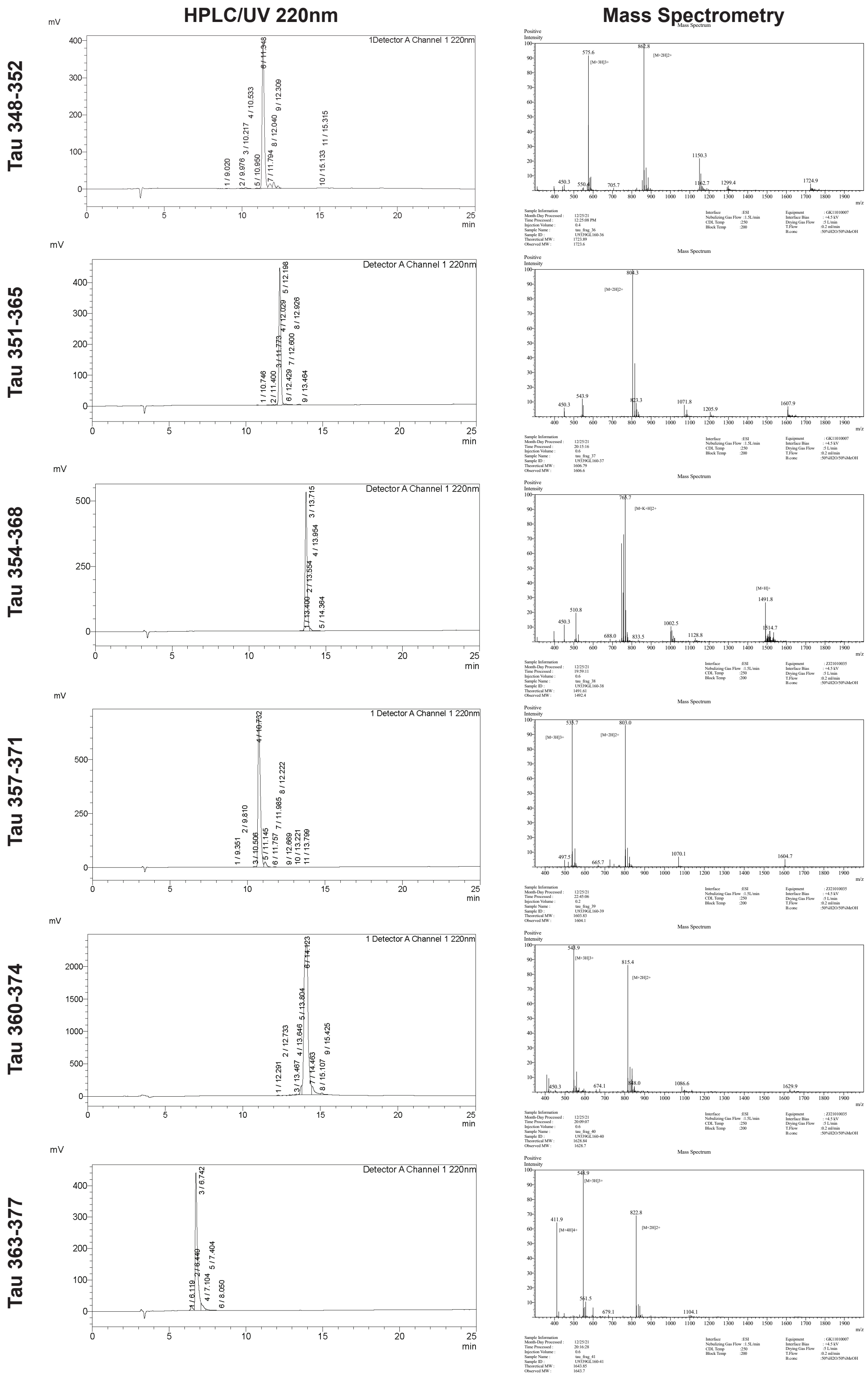

Supplementary Figure 16. Peptide quality control. HPLC chromatograms (left) and mass spectrometry (right) for peptides spanning tauRD residues 348-377.

Tau 243-257

Detector A Channel 1 220nm

Peak#	Ret. Time	Area	Height	Area%
1	7.263	4653	603	0.049
2	11.709	15663	1473	0.164
3	12.864	195457	25126	2.050
4	13.727	5653	362	0.059
5	14.139	31267	3173	0.328
6	14.463	102073	10785	1.071
7	14.692	180391	12178	1.892
8	15.347	8983780	780669	94.227
9	16.083	15289	793	0.160
Total		9534226	835162	100.000

Tau 252-266

Detector A Channel 1 220nm

Peak#	Ret. Time	Area	Height	Area%
1	5.360	20600	3228	1.788
2	11.291	5935	1016	0.515
3	12.232	1036905	123883	90.019
4	12.711	31454	3783	2.731
5	12.982	3984	697	0.346
6	13.132	52991	7783	4.600
Total		1151870	140390	100.000

Tau 261-275

Detector A Channel 1 220nm

Peak#	Ret. Time	Area	Height	Area%
1	9.588	5878	803	0.100
2	9.869	104039	13591	1.774
3	10.170	39025	3196	0.665
4	10.483	123898	9134	2.113
5	10.699	5519303	716666	94.107
6	10.938	72763	11677	1.241
Total		5864907	755066	100.000

Tau 270-284

Detector A Channel 1 220nm

Peak#	Ret. Time	Area	Height	Area%
1	12.624	5486	823	0.042
2	13.125	44177	7019	0.339
3	13.437	9946	1374	0.076
4	13.629	29687	7232	0.228
5	13.883	12711099	1276885	97.477
6	14.138	177988	22809	1.365
7	14.579	29143	1841	0.223
8	16.639	2201	270	0.017
9	17.460	9723	1226	0.075
10	18.187	6555	1007	0.050
11	19.910	11852	1620	0.091
12	21.883	1019	117	0.008
13	24.715	1179	141	0.009
Total		13040055	1322364	100.000

Tau 279-293

Detector A Channel 1 220nm

Peak#	Ret. Time	Area	Height	Area%
1	8.602	15813	1545	0.476
2	8.879	19435	1887	0.585
3	8.952	58197	11333	1.751
4	9.132	3180555	397529	95.706
5	9.429	8236	999	0.248
6	9.984	30920	2053	0.930
7	10.716	10086	919	0.303
Total		3323243	416265	100.000

Tau 288-302

Detector A Channel 1 220nm

Peak#	Ret. Time	Area	Height	Area%
1	8.249	62063	8458	0.217
2	8.427	2092385	364906	7.318
3	8.618	122720	28928	0.429
4	8.981	25849760	2656210	90.412
5	9.224	339389	38969	1.187
6	9.428	59637	10182	0.209
7	9.597	40598	7285	0.142
8	9.811	24537	4563	0.086
Total		28591088	3119502	100.000

Tau 297-311

Detector A Channel 1 220nm

Peak#	Ret. Time	Area	Height	Area%
1	7.865	33705	3804	0.778
2	13.814	25614	1965	0.591
3	14.137	28079	3735	0.648
4	14.394	4057736	527211	93.672
5	14.836	46737	5940	1.079
6	14.981	62926	8523	1.453
7	15.175	25396	3084	0.586
8	15.349	41197	4229	0.951
9	15.741	10470	983	0.242
Total		4331861	559475	100.000

Tau 246-260

Detector A Channel 1 220nm

Peak#	Ret. Time	Area	Height	Area%
1	12.892	23951	3492	0.172
2	13.096	9927	1496	0.071
3	15.338	15890	1321	0.114
4	15.774	205405	21669	1.475
5	16.176	72104	7347	0.518
6	16.506	13508263	1080776	97.014
7	16.929	54750	6670	0.393
8	17.517	33792	2066	0.243
Total		13924084	1124835	100.000

Tau 255-269

Detector A Channel 1 220nm

Peak#	Ret. Time	Area	Height	Area%
1	10.212	33322	6464	0.740
2	10.414	4333769	643520	96.183
3	10.563	77294	20776	1.715
4	10.707	61349	8715	1.362
Total		4505735	679475	100.000

Tau 264-278

Detector A Channel 1 220nm

Peak#	Ret. Time	Area	Height	Area%
1	8.357	3917	453	0.051
2	8.943	5744	861	0.075
3	9.350	2763	357	0.036
4	9.625	7265	805	0.094
5	9.800	8603	855	0.112
6	10.083	29320	2584	0.381
7	10.329	39136	6241	0.508
8	10.466	7160257	676591	92.989
9	10.829	254647	19765	3.307
10	11.217	65531	5970	0.851
11	11.514	122958	8225	1.597
Total		7700140	722706	100.000

Tau 273-287

Detector A Channel 1 220nm

Peak#	Ret. Time	Area	Height	Area%
1	10.259	2877	340	0.353
2	11.688	19054	4153	2.340
3	11.849	747645	97388	91.809
4	12.608	2972	250	0.365
5	12.867	10004	874	1.228
6	13.734	14290	1204	1.755
7	13.921	12674	1073	1.556
8	14.383	1358	169	0.167
9	14.552	3477	234	0.427
Total		814350	105684	100.000

Tau 282-296

Detector A Channel 1 220nm

Peak#	Ret. Time	Area	Height	Area%
1	12.600	1009	113	0.025
2	12.995	3736	578	0.092
3	13.258	17809	2745	0.438
4	13.365	26882	3961	0.662
5	13.603	3744724	579995	92.178
6	13.813	73160	7167	1.801
7	14.357	107435	9261	2.645
8	14.796	38317	4161	0.943
9	16.180	2481	372	0.061
10	16.616	27838	816	0.685
11	17.024	10879	820	0.268
12	19.937	8219	1100	0.202
Total		4062488	611090	100.000

Tau 291-305

Detector A Channel 1 220nm

Peak#	Ret. Time	Area	Height	Area%
1	7.491	217674	27535	2.731
2	7.774	7185571	659237	90.161
3	8.246	342445	33020	4.297
4	8.729	77543	6530	0.973
5	9.236	28254	1725	0.355
6	11.030	99172	6107	1.244
7	11.473	12821	1297	0.161
8	12.277	6240	864	0.078
Total		7969721	736314	100.000

Tau 300-314

Detector A Channel 1 220nm

Peak#	Ret. Time	Area	Height	Area%
1	12.831	5270	592	0.487
2	13.082	1018064	160636	94.138
3	13.536	20376	1502	1.884
4	13.771	4138	691	0.383
5	13.877	7145	960	0.661
6	13.983	3502	642	0.324
7	14.196	5883	810	0.544
8	14.420	5873	748	0.543
9	14.513	5293	680	0.489
10	14.764	3392	441	0.314
11	15.027	1121	146	0.104
12	15.186	1396	172	0.129
Total		1081454	168019	100.000

Tau 249-263

Detector A Channel 1 220nm

Peak#	Ret. Time	Area	Height	Area%
1	10.289	178417	16611	0.983
2	10.962	16812299	1409228	92.602
3	11.204	304210	33707	1.676
4	11.429	111203	9533	0.613
5	12.695	45781	3950	0.252
6	13.906	47476	3346	0.261
7	14.799	89117	7703	0.491
8	15.138	88147	8148	0.486
9	15.363	176171	13781	0.970
10	15.602	64456	9339	0.355
11	15.825	27649	4759	0.152
12	18.023	80490	12823	0.443
13	19.014	32858	4660	0.181
14	19.488	80380	7709	0.443
15	20.760	16805	1082	0.093
Total		18155458	1546378	100.000

Tau 258-272

Detector A Channel 1 220nm

Peak#	Ret. Time	Area	Height	Area%
1	10.588	43865	5555	1.351
2	10.739	3012352	379983	92.754
3	10.946	103787	11900	3.196
4	11.280	17400	2035	0.536
5	11.503	62186	3701	1.915
6	12.168	5360	507	0.165
7	12.343	2736	422	0.084
Total		3247686	404103	100.000

Tau 267-281

Detector A Channel 1 220nm

Peak#	Ret. Time	Area	Height	Area%
1	5.228	2725	452	0.076
2	7.284	19813	2478	0.555
3	7.515	3354702	307038	94.041
4	7.979	124385	11262	3.487
5	8.254	55490	3801	1.556
6	23.524	10146	1074	0.284
Total		3567260	326106	100.000

Tau 276-290

Detector A Channel 1 220nm

Peak#	Ret. Time	Area	Height	Area%
1	9.631	4050	554	0.164
2	10.931	1002	136	0.041
3	11.442	2223	282	0.090
4	11.571	2626	281	0.106
5	12.288	10458	1430	0.424
6	12.979	4850	859	0.197
7	13.149	2389728	292508	96.858
8	13.379	48289	8916	1.957
9	13.885	4023	376	0.163
Total		2467249	305342	100.000

Tau 285-299

Detector A Channel 1 220nm

Peak#	Ret. Time	Area	Height	Area%
1	7.994	44189	7466	2.187
2	8.129	24569	6181	1.216
3	8.283	1825882	270027	90.378
4	8.454	58233	12920	2.882
5	8.579	35572	5324	1.761
6	8.745	31833	4803	1.576
Total		2020279	306720	100.000

Tau 294-308

Detector A Channel 1 220nm

Peak#	Ret. Time	Area	Height	Area%
1	10.550	8966	1433	0.077
2	10.783	612647	104083	5.283
3	10.964	13046	2750	0.112
4	11.136	57397	8749	0.495
5	11.428	10596249	1343378	91.376
6	11.718	211219	33804	1.821
7	11.992	96801	13441	0.835
Total		11596325	1507638	100.000

Tau 303-317

Detector A Channel 1 220nm

Peak#	Ret. Time	Area	Height	Area%
1	13.567	2257	348	0.038
2	13.788	8673	3167	0.146
3	13.944	5840413	950749	98.172
4	14.156	97819	16699	1.644
Total		5949162	970964	100.000

Supplementary Figure 17. Peak tables for HPLC of peptides spanning tauRD residues 243-317.

Tau 306-320

Detector A Channel 1 220nm

Peak#	Ret. Time	Area	Height	Area%
1	13.091	8375	1509	0.050
2	13.853	5094	718	0.031
3	14.104	11342	1834	0.068
4	14.537	9612	1820	0.058
5	14.658	24016	4149	0.145
6	14.832	35083	6256	0.211
7	14.978	190166	28186	1.146
8	15.127	47329	7162	0.285
9	15.261	45867	6988	0.276
10	15.576	15603976	1687861	94.033
11	15.813	195841	29826	1.180
12	15.971	95693	14132	0.577
13	16.309	16026	2051	0.097
14	17.456	279117	35414	1.682
15	19.439	3412	261	0.021
16	19.907	18086	2474	0.109
17	21.885	3819	375	0.023

Tau 315-329

Detector A Channel 1 220nm

Peak#	Ret. Time	Area	Height	Area%
1	11.769	29202	2609	0.531
2	12.044	43040	3320	0.783
3	12.359	111459	22376	2.028
4	12.561	5116671	647405	93.104
5	12.804	36857	6767	0.671
6	13.083	47337	3773	0.861
7	13.253	111101	19279	2.022
Total		5495667	705528	100.000

Tau 324-338

Detector A Channel 1 220nm

Peak#	Ret. Time	Area	Height	Area%
1	7.923	140528	10247	1.704
2	8.225	8036909	820782	97.427
3	8.579	71687	6499	0.869
Total		8249123	837528	100.000

Tau 333-347

Detector A Channel 1 220nm

Peak#	Ret. Time	Area	Height	Area%
1	11.541	773147	124856	96.395
2	12.434	4633	167	0.578
3	17.736	1896	56	0.236
4	18.237	1423	51	0.177
5	19.756	2927	266	0.365
6	21.824	1218	165	0.152
7	22.425	2904	199	0.362
8	24.577	13908	591	1.734
Total		802057	126352	100.000

Tau 342-356

Detector A Channel 1 220nm

Peak#	Ret. Time	Area	Height	Area%
1	11.103	83150	2826	2.186
2	12.786	6444	1090	0.169
3	13.051	3920	740	0.103
4	13.274	3686236	440603	96.906
5	13.588	24175	4909	0.636
Total		3803925	450169	100.000

Tau 351-365

Detector A Channel 1 220nm

Peak#	Ret. Time	Area	Height	Area%
1	10.746	4778	772	0.125
2	11.400	5701	427	0.149
3	11.773	10428	1306	0.272
4	12.029	23020	2937	0.601
5	12.198	3667778	440358	95.720
6	12.429	28559	5106	0.745
7	12.600	37166	3434	0.970
8	12.926	20330	1316	0.531
9	13.464	34004	2605	0.887
Total		3831765	458261	100.000

Tau 360-374

Detector A Channel 1 220nm

Peak#	Ret. Time	Area	Height	Area%
1	12.291	57072	8814	0.123
2	12.733	15599	2682	0.034
3	13.467	159742	11313	0.345
4	13.646	35601	11840	0.077
5	13.804	868839	137329	1.876
6	14.123	43138269	2340086	93.137
7	14.463	1650290	130957	3.563
8	15.107	328414	29272	0.709
9	15.425	63060	6735	0.136
Total		46316886	2679027	100.000

Tau 309-323

Detector A Channel 1 220nm

Peak#	Ret. Time	Area	Height	Area%
1	14.671	4873	1084	0.212
2	14.839	36985	2700	1.607
3	15.270	73361	5657	3.188
4	15.574	2089875	290430	90.832
5	15.763	21579	4790	0.938
6	15.996	35612	3041	1.548
7	16.369	38534	1847	1.675
Total		2300820	309548	100.000

Tau 318-332

Detector A Channel 1 220nm

Peak#	Ret. Time	Area	Height	Area%
1	7.651	13267	1987	0.296
2	7.971	55955	5474	1.250
3	8.174	80518	7410	1.799
4	8.407	109354	13431	2.443
5	8.626	4162188	410986	92.990
6	9.088	54694	5952	1.222
Total		4475976	445241	100.000

Tau 327-341

Detector A Channel 1 220nm

Peak#	Ret. Time	Area	Height	Area%
1	12.291	57072	8814	0.123
2	12.733	15599	2682	0.034
3	13.467	159742	11313	0.345
4	13.646	35601	11840	0.077
5	13.804	868839	137329	1.876
6	14.123	43138269	2340086	93.137
7	14.463	1650290	130957	3.563
8	15.107	328414	29272	0.709
9	15.425	63060	6735	0.136
Total		46316886	2679027	100.000

Tau 336-350

Detector A Channel 1 220nm

Peak#	Ret. Time	Area	Height	Area%
1	12.295	21262	4156	0.238
2	14.288	74267	13447	0.830
3	15.335	284464	8303	3.179
4	16.488	8550656	996747	95.554
5	16.721	6277	1318	0.070
6	17.183	11547	1828	0.129
Total		8948472	1025799	100.000

Tau 345-359

Detector A Channel 1 220nm

Peak#	Ret. Time	Area	Height	Area%
1	12.956	27165	5002	0.423
2	13.104	49589	8456	0.772
3	13.292	6105723	836694	95.014
4	13.479	91793	24302	1.428
5	13.579	61418	8379	0.956
6	13.803	25479	4077	0.396
7	13.916	25534	4053	0.397
8	14.072	12469	2080	0.194
9	15.091	26946	4968	0.419
Total		6426116	898011	100.000

Tau 354-368

Detector A Channel 1 220nm

Peak#	Ret. Time	Area	Height	Area%
1	13.400	10207	1275	0.224
2	13.554	16457	2523	0.362
3	13.715	4432825	526579	97.422
4	13.954	72692	11882	1.598
5	14.364	17945	1482	0.394
Total		4550125	543741	100.000

Tau 363-377

Detector A Channel 1 220nm

Peak#	Ret. Time	Area	Height	Area%
1	6.119	7823	982	0.165
2	6.449	145739	16004	3.078
3	6.742	4275607	434795	90.290
4	7.104	229242	21545	4.841
5	7.404	63725	4759	1.346
6	8.050	13272	760	0.280
Total		4735409	478844	100.000

Tau 312-326

Detector A Channel 1 220nm

Peak#	Ret. Time	Area	Height	Area%
1	8.967	2582	558	0.084
2	10.228	28312	3958	0.917
3	12.456	63510	2450	2.058
4	13.098	2920697	251122	94.640
5	13.837	21783	1225	0.706
6	14.236	5948	957	0.193
7	14.440	43287	3332	1.403
Total		3086117	263600	100.000

Tau 321-335

Detector A Channel 1 220nm

Peak#	Ret. Time	Area	Height	Area%
1	10.289	178417	16611	0.983
2	10.962	16812299	1409228	92.602
3	11.204	304210	33707	1.676
4	11.429	111203	9533	0.613
5	12.695	45781	3950	0.252
6	13.906	47476	3346	0.261
7	14.799	89117	7703	0.491
8	15.138	88147	8148	0.486
9	15.363	176171	13781	0.970
10	15.602	64456	9339	0.355
11	15.825	27649	4759	0.152
12	18.023	80490	12823	0.443
13	19.014	32858	4660	0.181
14	19.488	80380	7709	0.443
15	20.760	16805	1082	0.093
Total		18155458	1546378	100.000

Tau 330-344

Detector A Channel 1 220nm

Peak#	Ret. Time	Area	Height	Area%
1	5.267	28812	4020	2.051
2	6.946	4611	410	0.328
3	7.144	9120	1168	0.649
4	7.413	1336976	142775	95.158
5	7.998	25482	1345	1.814
Total		1405002	149719	100.000

Tau 339-353

Detector A Channel 1 220nm

Peak#	Ret. Time	Area	Height	Area%
1	10.289	178417	16611	0.983
2	10.962	16812299	1409228	92.602
3	11.204	304210	33707	1.676
4	11.429	111203	9533	0.613
5	12.695	45781	3950	0.252
6	13.906	47476	3346	0.261
7	14.799	89117	7703	0.491
8	15.138	88147	8148	0.486
9	15.363	176171	13781	0.970
10	15.602	64456	9339	0.355
11	15.825	27649	4759	0.152
12	18.023	80490	12823	0.443
13	19.014	32858	4660	0.181
14	19.488	80380	7709	0.443
15	20.760	16805	1082	0.093
Total		18155458	1546378	100.000

Tau 348-362

Detector A Channel 1 220nm

Peak#	Ret. Time	Area	Height	Area%
1	9.020	3906	534	0.093
2	9.976	11833	1058	0.281
3	10.217	26864	2628	0.638
4	10.533	2093	310	0.050
5	10.950	9342	1202	0.222
6	11.348	3789659	388352	90.023
7	11.794	147500	12450	3.504
8	12.040	151580	16862	3.601
9	12.309	57328	6528	1.362
10	15.133	3167	348	0.075
11	15.315	6392	673	0.152
Total		4209664	430945	100.000

Tau 357-371

Detector A Channel 1 220nm

Peak#	Ret. Time	Area	Height	Area%
1	9.351	1527	175	0.020
2	9.810	4251	379	0.056
3	10.506	37724	3720	0.494
4	10.732	7312052	693033	95.673
5	11.145	259404	23696	3.394
6	11.757	14753	1413	0.193
7	11.985	2929	395	0.038
8	12.222	2479	277	0.032
9	12.669	3418	223	0.045
10	13.221	2280	155	0.030
11	13.799	1967	251	0.026
Total		7642784	723717	100.000

Supplementary Figure 18. Peak tables for HPLC of peptides spanning tauRD residues 306-377.

Supplementary Table 1. Basic information of the patients and disease stage. An informed consent for autopsy and scientific use of autopsy tissue with clinical information was granted from all subjects involved.

**Sample ID**	**Source**	**Age (years)**	**Sex**	**Neuropathological diagnosis**	**Clinical diagnosis**	**Braak NFT stage**	**Aβ phase**	**CERAD score**	**NIA-AA degree of AD pathology**	**Post Mortem Interval/hours**
AD1	UZ/KU Leuven	71	Female	AD	AD	VI	5	2	high	24
AD2	UZ/KU Leuven	87	Male	AD	AD	VI	5	2	high	12
AD3	UZ/KU Leuven	71	Male	AD	AD	V	5	3	high	12
AD4	University of British Columbia (UBC)	70	Female	AD	AD	VI	5	Freq	High	24
CBD	University of British Columbia (UBC)	70	Male	CBD	Behavioral variant FTD (bvFTD)	NA	3	Sparce	NA	24
PSP	University of British Columbia (UBC)	86	Female	PSP	PSP	NA	2	0	NA	>48
PiD	University of British Columbia (UBC)	66	Female	PiD	Primary Progressive Aphasia (PPA)	III	0	0	NA	>48

Supplementary Table 2. Data collection, refinement and validation statistics for the Tau-PAM4 cryo-EM datasets.

	Acetyl-PAM4 Type 1 (EMD-16876) (PDB-8oh2)	Fmoc-PAM4 Type 2 (EMD-16881) (PDB-8ohi)	Fmoc-PAM4 Type 3 (EMD-16883) (PDB-8ohp)	Fmoc-PAM4 Type 4 (EMD-16886) (PDB 8oi0)
**Data collection and processing**				
Magnification	130,000	130,000		
Voltage (kV)	300	300		
Detector	Falcon4	Falcon4		
Energy filter	Selectris	Selectris		
Electron exposure (e^{-}/Å^2)	39	32		
Exposure rate (e^{-}/pixel/s)	7.0	5.7		
Nominal defocus range (μm)	-1.4 to -2.6	-1.2 to -2.4		
Pixel size (Å)	0.95	0.94		
Movies collected	1,512	1,957		
Initial particle images (no.)	286,927	325,180		
Final particle images (no.)	17,820	11,255	11,319	14,404
Map resolution (Å)	2.6	2.8	2.7	2.9
FSC threshold	0.143	0.143	0.143	0.143
Map resolution range (Å)	2.6-4.6	2.7-5.4	2.6-5.9	2.9-5.7
Symmetry imposed	C1	C1	C1	C1
Helical parameters				
Helical twist (°)	359.32	358.55	358.95	358.92
Helical rise (Å)	4.86	4.80	4.80	4.80
Crossover distance (nm)	125	60	82	80
**Refinement**				
Initial model used (PDB code)	-	-	8ohi	8ohp
Map sharpening *B* factor (Å^2)	-35	-39	-50	-26
Cross correlation (masked)	0.83	0.84	0.80	0.82
Model resolution	2.2	2.1	2.3	2.6
FSC threshold	0.143	0.143	0.143	0.143
Model composition				
Non-hydrogen atoms	3702	2088	2784	2784
Protein residues total	468	234	312	312
Ligand/solvent molecules	30 (H_2O)	18 (fmoc)	24 (fmoc)	24 (fmoc)
Chains per helical layer	12	6	8	8
Helical layers modelled	3	3	3	3
B factors (Å^2)				
Protein	54	48	48	64
Ligand/solvent	36	26	35	48
R.m.s. deviations				
Bond lengths (Å)	0.007	0.006	0.006	0.007
Bond angles (°)	0.604	0.531	0.627	0.662
Validation				
MolProbity score	1.0	1.0	1.1	1.2
Clashscore	2.0	1.3	2.7	3.9
Poor rotamers (%)	0.0	1.4	0.0	0.0
Ramachandran plot				
Favored (%)	100.0	98.5	100.0	100.0
Allowed (%)	00.0	1.5	0.0	0.0
Disallowed (%)	0.0	0.0	0.0	0.0

Supplementary References

1 Leistner, C. *et al.* The in-tissue molecular architecture of β-amyloid pathology in the mammalian brain. *Nature Communications* **14**, 2833 (2023). https://doi.org:10.1038/s41467-023-38495-5
