# Supplementary material for: Local structural preferences in shaping tau amyloid polymorphism
Source: Nat Commun. 2024 Feb 3;15:1028. doi: 10.1038/s41467-024-45429-2 (PMC10838331; doi:10.1038/s41467-024-45429-2)
Supplement: Supplementary file 3 — Description of additional supplementary files [file 41467_2024_45429_MOESM3_ESM.pdf]

Description of additional supplementary files

Supplementary Movie 1 - Movie of PAM4 tomograms along the z-axis (bottom-up, from back to the front of fibrils), example 1

Supplementary Movie 2 - Movie of PAM4 tomograms along the z-axis (bottom-up, from back to the front of fibrils), example 1
