# Supplementary material for: Local structural preferences in shaping tau amyloid polymorphism
Source: Nat Commun. 2024 Feb 3;15:1028. doi: 10.1038/s41467-024-45429-2 (PMC10838331; doi:10.1038/s41467-024-45429-2)
Supplement: Supplementary file 6 — Reporting Summary [file 41467_2024_45429_MOESM6_ESM.pdf]

nature portfolio

Corresponding author(s): Frederic Rousseau
Joost Schymkowitz

Last updated by author(s): Dec 24, 2023

Reporting Summary

Nature Portfolio wishes to improve the reproducibility of the work that we publish. This form provides structure for consistency and transparency in reporting. For further information on Nature Portfolio policies, see our Editorial Policies and the Editorial Policy Checklist.

Statistics

For all statistical analyses, confirm that the following items are present in the figure legend, table legend, main text, or Methods section.

n/a	Confirmed	
☐	☒	The exact sample size (n) for each experimental group/condition, given as a discrete number and unit of measurement
☐	☒	A statement on whether measurements were taken from distinct samples or whether the same sample was measured repeatedly
☐	☒	The statistical test(s) used AND whether they are one- or two-sided<br>*Only common tests should be described solely by name; describe more complex techniques in the Methods section.*
☒	☐	A description of all covariates tested
☒	☐	A description of any assumptions or corrections, such as tests of normality and adjustment for multiple comparisons
☐	☒	A full description of the statistical parameters including central tendency (e.g. means) or other basic estimates (e.g. regression coefficient) AND variation (e.g. standard deviation) or associated estimates of uncertainty (e.g. confidence intervals)
☐	☒	For null hypothesis testing, the test statistic (e.g. F, t, r) with confidence intervals, effect sizes, degrees of freedom and P value noted<br>*Give P values as exact values whenever suitable.*
☒	☐	For Bayesian analysis, information on the choice of priors and Markov chain Monte Carlo settings
☒	☐	For hierarchical and complex designs, identification of the appropriate level for tests and full reporting of outcomes
☒	☐	Estimates of effect sizes (e.g. Cohen's d, Pearson's r), indicating how they were calculated

Our web collection on statistics for biologists contains articles on many of the points above.

Software and code

Policy information about availability of computer code

Data collection	Structural modeling and thermodynamic calculations were performed using FoldX 5 (as described in Van der Kant et al. 2022 Structure 30 (8):1178-1189). The method is available on Zenodo (https://zenodo.org/records/6393987). FTIR spectral acquisition and analysis was performed using the OPUS software (v8.5.29). ThT curves were monitored and analysed using software provided from BMG Labtech (OMEGA v5.11, MARS v3.33). The WALTZ and CORDAX webserver predictors were used to obtain aggregation profiles. EPU-3.0 (Thermofisher) was used to collect cryoEM data.
Data analysis	Adxv software for X-ray diffraction (v1.9.15). RELION(3.1), CTFFIND v4.14, Coot (0.9), Phenix v1.17.1, ChimeraX-1.3, PyMol-2.3.2 and the MolProbity webserver were used for cryoEM data processing, model building and refinement. High content screening analysis was performed using the Columbus Plus digital platform (PerkinElmer). Data plotting was performed using Prism 9. Yasara (21.12.19) was used for structural visualisations. Superpose v1.0 was used for structural alignments.

For manuscripts utilizing custom algorithms or software that are central to the research but not yet described in published literature, software must be made available to editors and reviewers. We strongly encourage code deposition in a community repository (e.g. GitHub). See the Nature Portfolio guidelines for submitting code & software for further information.

Data

Policy information about availability of data

All manuscripts must include a data availability statement. This statement should provide the following information, where applicable:

- Accession codes, unique identifiers, or web links for publicly available datasets
- A description of any restrictions on data availability
- For clinical datasets or third party data, please ensure that the statement adheres to our policy

The PAM4 amyloid fibril cryoEM maps and models for the four solved structures are deposited in the EMDB and PDB respectively with codes: EMD-16876[https://www.ebi.ac.uk/emdb/EMD-16876], PDB-8oh2[https://www.rcsb.org/structure/8oh2] (PAM4 Type 1); EMD-16881[https://www.ebi.ac.uk/emdb/EMD-16881], PDB-8ohi[https://www.rcsb.org/structure/8ohi] (PAM4 Type 2); EMD-16883[https://www.ebi.ac.uk/emdb/EMD-16883], PDB-8ohp[https://www.rcsb.org/structure/8ohp] (PAM4 Type 3); EMD-16886[https://www.ebi.ac.uk/emdb/EMD-16886], PDB-8oi0[https://www.rcsb.org/structure/8oi0] (PAM4 Type4). The following PDB files were used in this study: 5O3O[https://www.rcsb.org/structure/5o3o], 5O3L[https://www.rcsb.org/structure/5o3l], 5O3T[https://www.rcsb.org/structure/5o3t], 6HRE[https://www.rcsb.org/structure/6hre], 6HRF[https://www.rcsb.org/structure/6hrf], 7NRV[https://www.rcsb.org/structure/7nrv], 7NRX[https://www.rcsb.org/structure/7nrx], 7MKH[https://www.rcsb.org/structure/7mkh], 7NRQ[https://www.rcsb.org/structure/7nrq], 7NRS[https://www.rcsb.org/structure/7nrs], 7NRT[https://www.rcsb.org/structure/7nrt], 7MKF[https://www.rcsb.org/structure/7mkf], 7MKG[https://www.rcsb.org/structure/7mkg], 6NWP[https://www.rcsb.org/structure/6nwp], 6NWQ[https://www.rcsb.org/structure/6nwq], 7QJW[https://www.rcsb.org/structure/7qjw], 6TJX[https://www.rcsb.org/structure/6tjx], 6TJO[https://www.rcsb.org/structure/6tjo], 6VH7[https://www.rcsb.org/structure/6vh7], 6VHA[https://www.rcsb.org/structure/6vha], 7P6D[https://www.rcsb.org/structure/7p6d], 7P6E[https://www.rcsb.org/structure/7p6e], 6GX5[https://www.rcsb.org/structure/6gx5], 6QJH[https://www.rcsb.org/structure/6qjh], 6QJM[https://www.rcsb.org/structure/6qjm], 6QJP[https://www.rcsb.org/structure/6qjp], 6QJQ[https://www.rcsb.org/structure/6qjq], 7P65[https://www.rcsb.org/structure/7p65], 7P6A[https://www.rcsb.org/structure/7p6a], 7P6B[https://www.rcsb.org/structure/7p6b], 7P6C[https://www.rcsb.org/structure/7p6c], 7P66[https://www.rcsb.org/structure/7p66], 7P67[https://www.rcsb.org/structure/7p67], 7P68[https://www.rcsb.org/structure/7p68]. Source data are provided in this paper.

Research involving human participants, their data, or biological material

Policy information about studies with human participants or human data. See also policy information about sex, gender (identity/presentation), and sexual orientation and race, ethnicity and racism.

Reporting on sex and gender	Information provided in Supplementary Table 1. Self-reporting. No sex/gender-based (7 individuals, 3 male, 4 female) consideration and analysis was performed (small size).
Reporting on race, ethnicity, or other socially relevant groupings	NA
Population characteristics	Information provided in Supplementary Table 1. Samples were obtained from 7 individuals ranging from 66 to 87 years of age. Diagnosis: 4 individuals with AD, 1 individual with PSP, CBD, and Pick's Disease.
Recruitment	Brain tissue from autopsy cases was received from UZ/KU Leuven Biobank and University of British Columbia (UBC). For all samples an informed consent sheet for autopsy and scientific use has been signed either by the patient during life or by the next in kin after death.
Ethics oversight	The research involving human tissue samples was carried out under ethical approval by the UZ Leuven ethical committee (Leuven/Belgium; File-No. S63759).

Note that full information on the approval of the study protocol must also be provided in the manuscript.

Field-specific reporting

Please select the one below that is the best fit for your research. If you are not sure, read the appropriate sections before making your selection.

☒ Life sciences ☐ Behavioural & social sciences ☐ Ecological, evolutionary & environmental sciences

For a reference copy of the document with all sections, see nature.com/documents/nr-reporting-summary-flat.pdf

Life sciences study design

All studies must disclose on these points even when the disclosure is negative.

Sample size	Sample size for experiments performed with patient samples was predetermined based on sample availability. Experiments with PSP, CBD and PiD patient extracts were performed in two independent repeats due to sample availability. All other in vitro and in cellulo experiments were performed at least with n=3. A minimum of 3 independent repeats was required to evaluate statistical significance. Details regarding sample sizes and statistical tests of all experiments are provided in the corresponding figure legends.
Data exclusions	No data were excluded in this study.
Replication	Experiments were performed in three or more independent replicates (PSP, CBD, PiD patient extracts used in duplicates to minimise sample consumption) to verify reproducibility and support statistical analysis. All replicates included in the main manuscript.

Randomization	Not relevant for this study. All experimental conditions were the same for all biological and technical replicates.
Blinding	No blinding, as the researchers needed to be fully aware of the samples involved to properly interpret the results.

Reporting for specific materials, systems and methods

We require information from authors about some types of materials, experimental systems and methods used in many studies. Here, indicate whether each material, system or method listed is relevant to your study. If you are not sure if a list item applies to your research, read the appropriate section before selecting a response.

Materials & experimental systems

n/a	Involved in the study	
x		Antibodies
	x	Eukaryotic cell lines
x		Palaeontology and archaeology
x		Animals and other organisms
x		Clinical data
x		Dual use research of concern
x		Plants

Methods

n/a	Involved in the study	
x		ChIP-seq
x		Flow cytometry
x		MRI-based neuroimaging

Eukaryotic cell lines

Policy information about cell lines and Sex and Gender in Research

Cell line source(s)	Human embryonic kindey cells (293T, ATCC CRL-3216) were obtained from ATCC.
Authentication	No authentication was performed in the lab. The cell lines were obtained from a commercial source and were already been authenticated using the companies' own quality control policies.
Mycoplasma contamination	Cells tested negative for mycoplasma.
Commonly misidentified lines (See ICLAC register)	None.

Plants

Seed stocks	*Report on the source of all seed stocks or other plant material used. If applicable, state the seed stock centre and catalogue number. If plant specimens were collected from the field, describe the collection location, date and sampling procedures.*
Novel plant genotypes	*Describe the methods by which all novel plant genotypes were produced. This includes those generated by transgenic approaches, gene editing, chemical/radiation-based mutagenesis and hybridization. For transgenic lines, describe the transformation method, the number of independent lines analyzed and the generation upon which experiments were performed. For gene-edited lines, describe the editor used, the endogenous sequence targeted for editing, the targeting guide RNA sequence (if applicable) and how the editor was applied.*
Authentication	*Describe any authentication procedures for each seed stock used or novel genotype generated. Describe any experiments used to assess the effect of a mutation and, where applicable, how potential secondary effects (e.g. second site T-DNA insertions, mosiacism, off-target gene editing) were examined.*
